# Quantitative Study of the Effect of Tissue Microstructure on Contraction in a Computational Model of Rat Left Ventricle

**DOI:** 10.1371/journal.pone.0092792

**Published:** 2014-04-02

**Authors:** Valentina Carapella, Rafel Bordas, Pras Pathmanathan, Maelene Lohezic, Jurgen E. Schneider, Peter Kohl, Kevin Burrage, Vicente Grau

**Affiliations:** 1 Department of Computer Science, University of Oxford, Oxford, United Kingdom; 2 Radcliffe Department of Medicine, Division of Cardiovascular Medicine, University of Oxford, Oxford, United Kingdom; 3 National Heart and Lung Institute, Imperial College, London, United Kingdom; 4 Institute of Biomedical Engineering, Department of Engineering Science, University of Oxford, Oxford, United Kingdom; Scuola Superiore Sant'Anna, Italy

## Abstract

Tissue microstructure, in particular the alignment of myocytes (fibre direction) and their lateral organisation into sheets, is fundamental to cardiac function. We studied the effect of microstructure on contraction in a computational model of rat left ventricular electromechanics. Different fibre models, globally rule-based or locally optimised to DT-MRI data, were compared, in order to understand whether a subject-specific fibre model would enhance the predictive power of our model with respect to the global ones. We also studied the impact of sheets on ventricular deformation by comparing: (a) a transversely isotropic versus an orthotropic material law and (b) a linear model with a bimodal model of sheet transmural variation. We estimated ejection fraction, wall thickening and base-to-apex shortening and compared them with measures from cine-MRI. We also evaluated Lagrangian strains as local metrics of cardiac deformation. Our results show that the subject-specific fibre model provides little improvement in the metric predictions with respect to global fibre models while material orthotropy allows closer agreement with measures than transverse isotropy. Nonetheless, the impact of sheets in our model is smaller than that of fibres. We conclude that further investigation of the modelling of sheet dynamics is necessary to fully understand the impact of tissue structure on cardiac deformation.

## Introduction

Cardiac tissue is arranged into a complex architecture. Due to their elongated shape, myocytes are highly anisotropic and their local orientation in cardiac tissue is usually called the fibre direction [1]. Furthermore, the extracellular connective tissue matrix that surrounds myocytes groups them into layers 4-6 cells thick [2], separated by cleavage planes, forming a branching laminar architecture. Such layers are commonly referred to as sheets. Although there is general agreement about the local alignment of cells and their organisation into sheets, disagreement remains on their exact characteristics in beating hearts, their alteration due to pathology and in general their degree of contribution to cardiac function [3]. Tissue microstructure contributes to the heterogeneity of tissue conductivity and therefore directly affects electrical wave propagation, with dramatic consequences in the case of pathology. Regarding mechanics, the importance of taking into account fibre and sheet orientation in the definition of passive and active behaviour of cardiac tissue has already been highlighted [4]. It is along the fibre direction that electrical excitation propagates fastest and that the principal component of contraction takes place. When included in an electromechanical model, fibres are often mathematically prescribed [5]. Recent advances in imaging technology have made it possible to measure local microstructure from data such as *ex vivo* diffusion tensor MRI (DT-MRI). The primary eigenvector of the diffusion tensor has been shown to correlate with the fibre direction in the tissue [6]. Electromechanical models based on DT-MRI data suggest that fibres are a dynamical system in cardiac structure, undergoing adaptation that minimises shear strains, behaviour that can have important consequences in tissue remodelling in pathology [7]. The importance of local myocyte orientation in cardiac function, both in health and disease, has promoted considerable effort in recent years towards subject-specific modelling of cardiac electromechanics [8], [9], [10]. Pathological conditions such as dyssynchrony [11] have been investigated, with realistic ventricular to whole-organ geometries, combined with fibre orientations obtained from imaging [10]. The results are promising, but it is an open question as to whether current electromechanical models are becoming a useful support tool in clinical decision-making.

**Figure 1:**
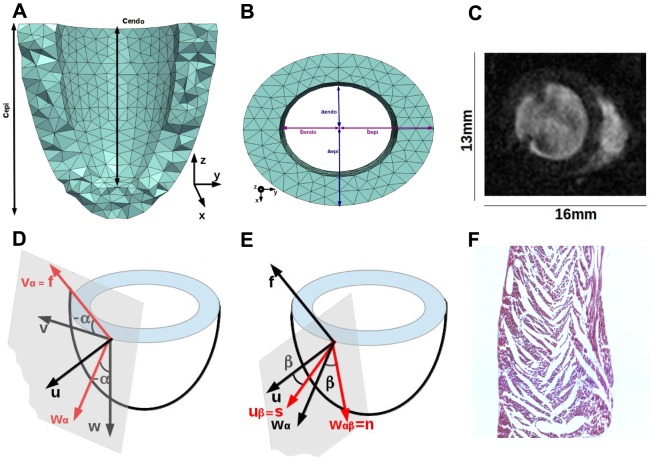
Modelling ventricular geometry and tissue structure. (a) The ellipsoidal mesh seen in a transmural-longitudinal cut. (b) Basal slice of the mesh cut parallel to the short axis. (c) Midventricular slice from rat cine-MRI at beginning of contraction. (d)-(e) Helix, and sheet angle definition. (f) Image of transmural histological sample highlighting the sheets.

The laminar structure of myocardial tissue is also important for cardiac electromechanics. It has been established that sheets undergo shortening in the fibre direction, extension in the in-sheet/cross-fibre plane, thickening and shear, whose regional variability and transmural distribution are important for the distribution of strain and stress throughout the ventricle during cardiac motion [12]. More recently, changes in sheet intersection angles have been identified to occur during the cycle of contraction and relaxation [13]. These sheet dynamics are thought to form one of the underlying mechanisms to explain wall-thickening during contraction [14]. Sheets have been modelled as continuously varying transmurally [2], although more recent experimental findings suggest that the transmural distribution of sheets is more complex and discontinuous [13], see for example Figure 1(f). Sheet orientations directly obtained from histological data, usually from a small number of ventricular locations only, have been employed in computational studies of the effect of tissue microstructure on electrophysiology [15], but in general the importance of sheets has not been fully acknowledged in the cardiac modelling community in spite of the experimental evidence.

Two motivations underlie our study. First, we aim to understand whether the use of subject-specific fibres would enhance the predictive power of our model, or whether it would be as informative as simple globally prescribed fibres within our modelling framework. To this purpose, globally defined rule-based fibres were compared with a subject-specific model of fibres that is locally optimised in order to minimise the discrepancy with DT-MRI data [5].

Our second motivation is the exploration of the often underestimated importance of sheet dynamics in the common electromechanical models. We want to test the impact of sheet orientation in the mechanics of the left ventricle. This was achieved by (a) comparing transverse isotropic with orthotropic material laws for cardiac tissue and (b) under the orthotropic material properties, testing two global models of transmural variation of the sheet orientation, namely a linear and a bimodal model. The aim of the latter comparison was to see whether the latest findings regarding the discontinuous multiple-population distribution of sheets [13] (mimicked by the bimodal model) can improve the accuracy of the electromechanical model, as opposed to the standard smooth variation of sheet orientation [2] (the linear model).

In order to quantify the effect of the different models of fibre and sheet orientation on contraction, global and local metrics were considered. The global metrics are ejection fraction, wall thickening, and shortening in the base-to-apex direction. We compared their predicted values with those obtained from rat cine-MRI data. The local metrics considered are the Lagrangian circumferential strain, as a measure of contraction, circumferential-radial shear strain, as a measure of the relative twist of sub-endocardial and sub-epicardial layers, and circumferential-longitudinal shear strain, as a measure of torsion [16].

A preliminary study of the effect of fibre direction and material orthotropy on contraction had been performed with a simplified electromechanical model (without pressure boundary conditions) [17]. The current study represents a significant extension in our model development, to further the study of the impact of sheets and the analysis of the local effect of tissue structure on deformation. The contribution of the study is twofold. First, we show that the fibre model affects significantly the prediction of the metrics considered. Therefore, it is important to choose the most suitable fibre setting. On the other hand, our results question the efficacy of using personalised myocyte orientations when compared to the limitations of state-of-the-art electromechanical models, since they do not seem to provide substantial improvements, both in terms of global and local measures, compared to simpler global rule-based fibre definitions, at least for the healthy left ventricle of rat. Secondly, we highlight the need for further development of models of sheets. On the one hand, the introduction of sheet orientation in the model by switching from transverse isotropy to orthotropy shows a small but consistent improvement in the metrics predictions. On the other hand, we found that, although experimentally more realistic, the bimodal model for sheet orientation did not improve and in some cases counteracted the effect of introducing material orthotropy. We believe that such an important structural change should affect more evidently the predicted deformation patterns. Given the results presented in this study, we conclude that our current electromechanical modelling approach may not be able to successfully make use of subject-specific tissue structure, with all its additional complexity and computational cost, unless a better understanding of the biological roles, and the computational modelling of sheets and their mechanical properties is achieved.

## Materials and Methods

No new animal experiment was required for the results presented in this paper. The images used come from a dataset previously published and for which the ethics statement was duly reported [18]. However, with respect to those experiments, the authors confirm that all animal work was conducted in accordance with Schedule 1 of the UK Home Office Guidance on the Operation of Animals (Scientific Procedures) Act of 1986, and was approved by the Oxford University ethical review board.

### MRI dataset

*In vivo* cine-MRI and subsequent *ex vivo* DT-MRI of rat hearts were acquired on a 9.4T (400 MHz) MR system (Agilent, Santa Clara, CA, USA). The cine-MRI dataset used a 2D multi-frame gradient echo sequence, and consisted of a stack of eight contiguous short-axis slices covering the heart from apex to the atrioventricular separation. Each short-axis slice comprised of 32–36 frames acquired throughout the cardiac cycle with an in-plane resolution of 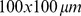 and a slice thickness of 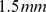. A short-axis view in end-diastole is shown in Figure 1(c). The DT-MRI images where subsequently acquired on excised rat hearts, fixed either in resting state or in contracture and embedded in agarose, at an isotropic resolution of 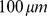 as described in [18].

### Geometrical mesh for rat left ventricle

We approximate the left ventricle with a thick-walled truncated ellipsoid. A first coarse tetrahedral mesh was generated with semi-axes and wall thickness estimated from the end-diastolic frame of one of the cine-MRI rat scans described (Figure 1(c)), which we took as the reference mesh (Figures 1(a) –1(b)). In order to estimate the shape of a mesh representative of the left ventricle in absence of blood pressure (called pre-P mesh), an iterative approach was applied. The reference mesh was shrunk by applying a uniform scaling matrix to give the pre-P mesh, which was then allowed to expand passively under a constant pressure of 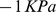 applied to the endocardial surface to give the post-P mesh (the negative sign means that the pressure is applied inwards along the normal to the surface). This step was repeated for several iterations adjusting the scaling factor until we obtained an optimal pre-P mesh producing a post-P mechanics mesh close to the reference mesh. Details about the pre-P and post-P meshes are given in Table 1. The mechanics mesh for the electromechanical simulations is the pre-P mesh resulting from the optimisation performed, and has quadratic elements. The mesh for the electrophysiological component is instead obtained by refining the post-P mechanics mesh and has approximately 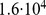 linear tetrahedral elements with a mean edge length of 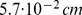. All meshes were generated with the open-source 3D finite element grid generator **Gmsh** [19].

**Table 1:** Mechanical mesh properties.

	pre-P mesh	post-P mesh
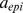	0.34	0.40
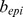	0.48	0.48
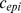	0.95	0.99
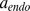	0.16	0.26
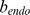	0.27	0.3
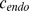	0.79	0.84
mean wall thickness	0.18	0.16
Cavity volume ( 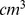 )	0.07	0.132
Tissue volume ( 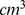 )	0.267	0.267
Mean edge length	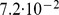	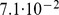
No. of elements	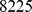	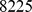

Mechanical mesh properties before (pre-P) and after (post-P) passive expansion under pressure. Length unit is 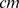. See Figures 1(a) –1(b) for reference to the ellipsoid parameters.

### Tissue structure definition

Tissue structure is defined at the centroid of each element of the mechanical mesh by assigning fibre, sheet and sheet-normal directions. This orthonormal local system of coordinates is assigned according to a well-established approach [2]. First, the orthonormal system of material coordinates (radial 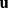, circumferential 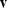 and longitudinal 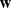 directions) for the centroid of a given element is identified. A first rotation about the radial direction 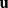 is performed, that transforms the vectors 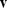 and 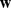 into 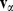 and 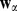. The rotation angle 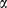 is the *helix angle* that defines the fibre direction 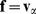. The second step consists of a rotation about the fibre direction 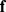 of the angle 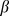, the *sheet angle*. The vector 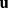 is rotated to 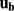 that represents the sheet direction 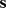, while the vector 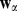 is rotated into 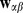 that gives the sheet-normal direction 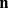. Figures 1(d) and 1(e) illustrate the first and second step, respectively.

Different models for the fibre and sheet orientation are introduced in this tissue structure generation framework by modelling the transmural variation of the helix and sheet angle as described below.

#### Models of fibre direction

**Figure 2:**
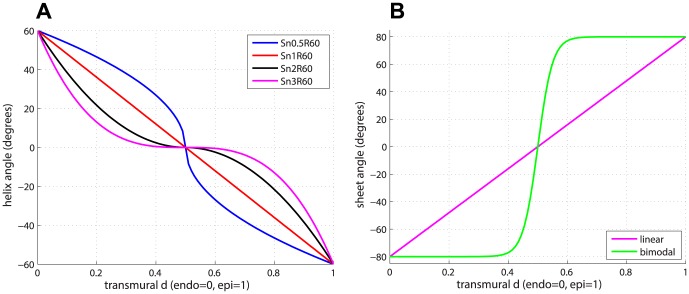
Helix and sheet angle variation. (a) Transmural models of the helix angle 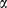 for four of the global rule-based fibre models. (b) Transmural models of sheet angle 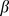.

A set of rule-based globally defined fibres were generated by assigning to each element of the mesh a value of the helix angle 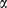, defined as the function 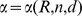 and calculated by eqn. (1)
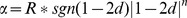
(1)where 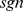 stands for the sign function, 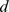 is the normalised transmural depth, varying from 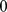 to 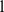 from endocardium to epicardium, 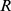 is the transmural rotation, i.e. the maximum absolute value of 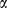 allowed by the model, which will span between 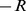 at epicardium and 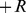 and endocardium. Finally, the exponent 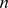 modulates the transmural variation of 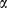, so for 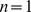 it will be linear, 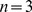 cubic. This is a standard approach for globally prescribed fibres introduced by Streeter et al. [1]. Four values of 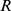 were tested, 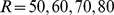 degrees, spanning the range of values found experimentally [5]. For each value of 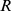, four values of the exponent 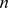 were considered, 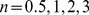, for a total of 16 globally defined fibre models. In the rest of the paper, these models are referred to as Sn
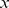
R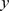, where S stands for Streeter-based and n
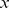
R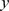 refers to the value of 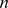 and 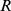 used. For example, the fibre model Sn0.5R70 is obtained by imposing 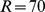 and 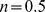 in eqn. (1). Figure 2(a) shows the transmural variation of 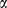 for a subset of these fibre models.

The global fibre models are compared with a subject-specific fibre model developed by Karadag et al. [5]. This is based on a local optimisation of 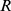 and 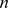 carried out on 6 subjects from the DT-MRI rat dataset previously described. The cine-MRI rat scan used to generate the mesh comes from one of the subjects from this set. The left ventricle was divided into 16 regions, an adaptation of the 17-region classification of the American Heart Association [20], and on each of them eqn. (1) was fitted in order to minimise the angular difference with the data. The result is a 16-regions model of fibres angles, with a single couple 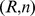 obtained for each region. We refer to this model as Karadag.

#### Models of sheet orientation

The standard approach to the modelling of sheet orientation is that of a smooth variation in the transmural direction [2]. Recent experiments question this modelling approach showing the presence in the tissue of sheet populations with opposing orientation angles [3] and a strong level of discontinuity [13]. In order to compare the effect of the two different approaches, we have compared a simple linear transmural variation of the sheet angle 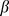 with a bimodal model. The linear model for 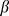 goes from 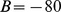 at endocardium to 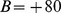 at epicardium, and is described by 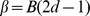. The bimodal model consists of a continuous approximation obtained by means of the sigmoidal function in eqn. (2)
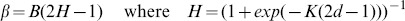
(2)where 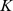 is a parameter affecting the steepness of the sigmoidal function. We chose 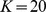 in order to have a distinct but smooth step in the trace. Figure 2(b) shows the transmural variation of the sheet angle 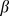 for the two models used in this study. In the following, unless it is specified, the linear model for the sheet angle is used.

### Electromechanical model

#### Model of electrophysiology

At the tissue level the monodomain equations (eqn. (3) and (4)) for electrical wave propagation are solved,
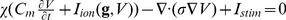
(3)

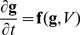
(4)where 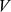 is the transmembrane voltage (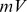), 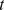 is time (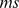), 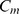 is the membrane capacitance (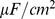), 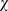 is the surface area-to-volume ratio (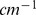), 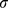 is the effective conductivity (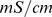), a matrix encoding the conductivity along the fibre, sheet and sheet-normal directions. The function 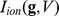 is the ionic current provided by the cell model (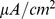) and 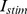 is the external stimulus (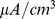). The vector 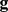 represents the set of state variables forming the cell electrophysiological model, whose temporal variation is regulated by the ordinary differential equation (ODE) system in eqn. (4). The model of cellular electrophysiology is that of Wang and Sobie [21]. Table 2 provides the parameters values we used for eqn. (3). The value of the surface area-to-volume ratio 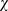 is typical for rat myocytes [22]. The conductivities were adjusted in order to ensure that the resulting conduction velocity in the fibre and cross-fibre directions was within experimental range, with ratio 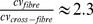 [23]. Cubic wedge simulations of electrophysiology with straight fibres parallel to one of the coordinates axes were used for this purpose.

**Table 2:** Parameters of the monodomain equation (eq. (3)).

Parameter	Value(s)
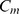	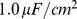
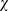	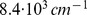
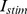	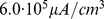
stim duration	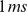
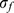	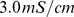
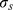	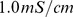
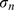	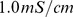

#### Model of mechanical behaviour

The mechanical component is coupled to the electrical component via the transmembrane voltage. At the tissue level, the mechanical behaviour is modelled according to finite elasticity theory. Let us consider two systems of Cartesian coordinates, a reference system defined on the undeformed body, 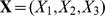, and a second system representing the deformed state, 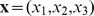. The components of the *deformation gradient tensor* 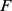, which describes the transformation from the undeformed state to deformed one, can be written as 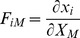. The *Green deformation tensor* 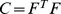, i.e. 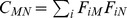, describes the deformation in the undeformed coordinate system. Finally, the *Lagrangian strain tensor* is 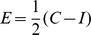, where 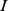 is the identity matrix.

The 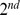 Piola-Kirchoff stress tensor 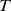 for the total stress produced by the tissue is the sum 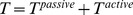 of the stress due to the tissue passive response, 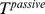, and active contraction, 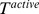. The constitutive relation between passive stress and strain is given by 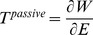, where 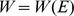 is the exponential strain-energy function used by Usyk et al. [4] and given in eqn. (5),
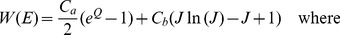
(5)

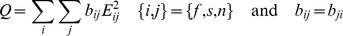
where 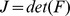, 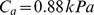 is a stiffness constant and 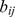 for 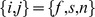 are stiffness parameters. The parameter 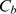 regulates the level of compressibility allowed by the material law: the higher the value the closer it is to being incompressible. After a preliminary analysis of the effect 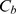 on the overall compressibility of the tissue we chose 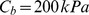, since it ensures a negligible volume reduction 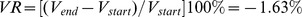 between beginning and end of contraction. Furthermore the distribution of the Jacobian of the deformation 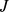 over the mesh elements between beginning and end of contraction has an average 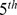
–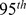 percentile range of 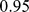
–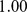, confirming that the model is nearly incompressible.

Two models of material passive behaviour were tested representing transversely isotropic and orthotropic behaviour. The stiffness parameters for the orthotropic case are taken from [4]. The parameters for the transversely isotropic law are obtained by averaging the relevant parameters of the orthotropic law. The parameter values for both laws are reported in Table 3.

**Table 3:** Stiffness parameters values for the material law (eq. (5)).

Parameter	transverse isotropy	orthotropy
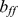	6.0	6.0
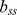	4.3	7.0
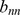	4.3	3.0
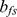	7.5	12.0
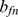	7.5	3.0
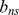	4.3	3.0

The active stress at the tissue level, 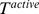, depends on the active tension generated at the cell level. The cell contraction model we used was originally developed by Kerkchoffs [24] for canine myocytes. The model parameters associated with the mechanical activation time in the twitch component of the model, (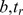 and 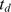), were reduced by 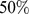 in order to get a shorter contraction duration (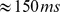), more compatible with the rat cardiac cycle frequency, while still generating an analogous amount of active tension (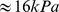 at peak contraction) in that shorter interval. Finally, the total stress 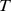 is required to satisfy the conservation of momentum as shown in eq. (6)
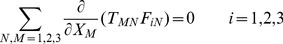
(6)where the effect of inertia and gravity is neglected. The system is solved with appropriate Dirichlet boundary conditions for displacement and Neumann boundary conditions for tractions, in this case the blood cavity pressure onto the endocardial surface, which is defined as follows.

#### Cavity pressure trace

**Figure 3:**
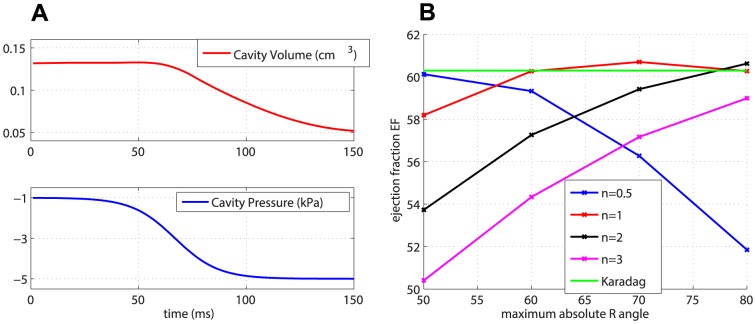
Pressure and volume traces and global metric 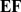. (a) Pressure trace and resulting cavity volume trace for simulation with fibre Sn1R70 and transverse isotropy. (b) 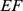 for the different fibre models, with transversely isotropic material law.

The temporal evolution of the intra-cavital blood pressure is approximated by means of a sigmoidal function ranging from 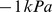 to 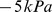, where the negative sign is due to the fact the pressure is applied in the inward direction along the normal to the surface. The range of pressure applied is significantly lower than that typically measured in the rat left ventricle, which reaches approximately 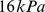 (or more, for example during physical activity) at end-systole [25]. This is a known limitation of some contraction models and follows existing computational modelling literature [26]. However, the chosen pressure trace ensures that the left ventricle undergoes first an isovolumic contraction and then an ejection phase. Figure 3(a) shows the pressure trace with an example of the resulting cavity volume.

### Electromechanical simulations

All simulations were run using **Chaste**, an open-source simulation package for computational biology developed in our group (http://www.cs.ox.ac.uk/chaste) [27]. The simulation duration is 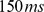 which is approximately the time at which full contraction is achieved. The timesteps used for solving the electrophysiology are 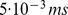 for the cell model (eq. (4)) and 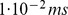 for the monodomain equation, while for the mechanics they are 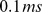 for the contraction model and 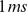 for eq. (6). The numerical scheme used has been described in previous work [28]. A single electrical stimulus of 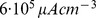 intensity is applied to the apical region, identified as the lower 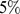 of the left ventricle. This type of activation region is commonly used in experiments with Langendorff-perfused hearts, and it was chosen to mimic the apex-to-base direction of the electrical activation in the left ventricle. Dirichlet boundary conditions fix the nodes at the base of the ventricle in the long-axis direction, so that they are allowed to move in the short-axis plane.

### Global and local metrics

The global measures used to evaluate the simulations are: ejection fraction 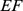, wall thickening 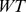 and shortening in the base-to-apex direction 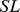. All the quantities are computed by comparing the state of the mesh at the beginning (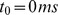) and end (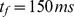) of contraction. 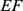 is calculated as the relative difference between the mesh cavity volume 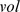 at 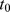 and 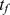, 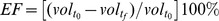. 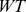 is the difference in wall-thickness 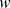, 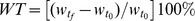, where 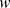 at a given time is obtained by averaging four measures of wall-thickness taken symmetrically onto a short-axis slice in the midventricular region (at approximately 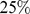 of the long axis from the base). We chose this specific short-axis slice to compare with the cine images, where 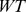 is more easily measured in the midventricular region. 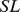 is calculated by comparing the 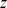 coordinate of the epicardial apical node along the 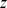 axis (which coincides with the long axis of the left ventricle, see Figure 1(a)) at 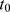 and 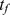, 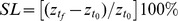. This is an adequate measure of the shortening along the base-to-apex direction since the base is kept fixed in the model and the apex moves almost exclusively along the long axis (of course, *in situ*, the movement is in the opposite direction, i.e. the atrio-ventricular valve plane moves ‘downwards’ in the direction of the apex, but in terms of changes in apico-basal length, this parameter offers a satisfactory approximation within the modelling framework used). Here 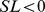 represents shortening, while 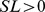 represents elongation of the ventricle during contraction.

In order to compare the predicted values with experimental data, we measured these metrics on the cine-MRI dataset used for building the ellipsoidal mesh by applying analogous methods. The measured values are 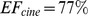, 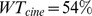 which are consistent with values in the literature [29], and 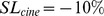.

As local metrics we considered the Lagrangian circumferential strain 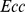, and two shear strains, the circumferential-longitudinal shear strain 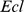, and the circumferential-radial shear strain 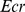. The Lagrangian strain tensor 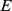 in the Cartesian system is obtained from the deformation tensor 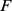 calculated during the simulation, that was sampled every 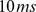. Then the conversion to cylindrical coordinates was applied. We could not compare these strain predictions directly with data, but they show good agreement with results in the literature as further explained in the Discussion.

## Results

In this section we present the results of the series of electromechanical simulations we performed in order to (a) compare sixteen globally defined fibre models (Sn
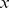
R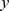 for 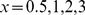 and 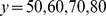) against one subject-specific fibre model (Karadag), and (b) evaluate the effect of introducing sheets by comparing transverse isotropy to orthotropy and with linear and bimodal sheet models. The results are grouped into global and local measures.

### Global measures

The global measures considered in this study are 1) ejection fraction 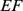, 2) wall thickening 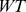, and 3) shortening in the long-axis direction 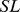. The variation in 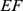 due to the different fibre models is shown in Figure 3(b), with the transversely isotropic material law. 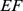 is plotted vs 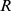, and the global fibre models Sn
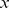
R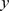 are grouped according to the exponent 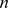 in order to highlight the pattern of 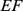 with increasing values of 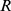 for each value of the exponent 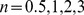. The single value of 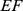 for the Karadag fibres is represented as an horizontal line. The predicted 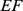 is overall in the range 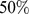
–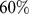, which can be considered physiological [30], but which is still lower than the measured value 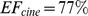. Among the global fibre models, for 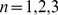 increasing values of the angle 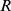 and decreasing values of the exponent 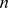 raise the value of 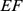. The reverse holds for 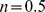, for which the increase in the maximum angle 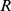 causes a rapid decrease of the 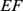. A possible explanation for this downward trend is provided in the Discussion. The subject-specific Karadag model gives one of the highest values of 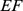 together with the global models Sn
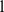
R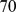 and Sn
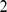
R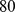.

**Figure 4:**
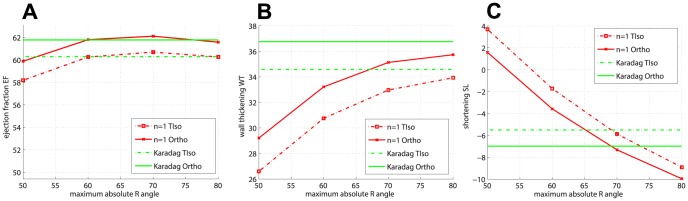
Material orthotropy vs transverse isotropy. Effect of introducing sheets by swicthing from transverse isotropy to orthotropy. (a) 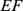 (b) 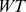 and (c) 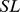 for global fibre models (with 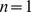) and Karadag model.

Changing from the transversely isotropic to the orthotropic material law (with the linear sheet model) has the general effect of increasing the 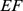 value for all the fibre models, with an average increase of 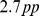 (percentage points), resulting in 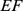 values in the range 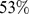
–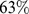, i.e. still below the measured value. Figure 4(a) shows the comparison of 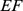 between transversely isotropic and orthotropic material laws with a linear sheet model for the simulations with global fibres with 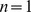 and Karadag fibres. Similar results are obtained for the other values of 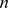. Within the orthotropic case, the effect of using a linear or a bimodal sheet model has the interesting effect of producing predictions much closer to those obtained under transverse isotropy than to orthotropy with the linear sheet model, but in general with a less consistent pattern across the fibre models (and therefore this is not shown).

**Figure 5:**
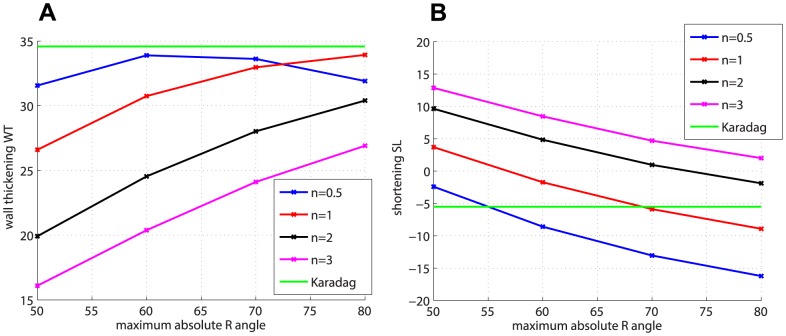
Global metrics 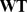 and 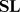. (a) 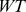 and (b) 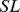 for the different fibre models, with transversely isotropic material law.

For 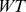, Figure 5(a) represents the wall thickening 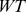 vs 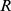, with the transversely isotropic material law. For the global fibre models, we observe the same trend as with 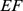, i.e. for increasing values of 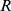 and decreasing values of 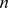 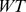 increases. Karadag fibres still produce one of the highest values of 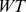, but some global fibres models also give good results. The use of the orthotropic material law with the linear sheet model further increases the value of 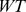 for every fibre model, as shown in Figure 4(b), with an average increase of 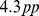. The effect of varying the sheet model from linear to bimodal produces again values closer to the transversely isotropic case. Overall the range of variability for 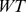 is 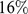
–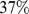, with only some fibre models giving values close to the physiological range. In any case there is a clear discrepancy between the predicted 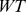 values and that measured on cine-MRI, 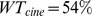.

The shortening in the base-to-apex direction (negative values of 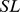) is also substantially affected by the fibre type and material law, ranging from 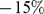 to 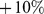. Figure 5(b) shows 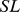 vs 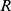, with the transversely isotropic material law. Within the global fibre models Sn
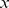
R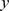, high values of the exponent 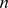 combined with low values of 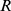 produce lengthening (positive values of 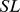), whereas there is increasing shortening for low values of 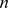 and increasing values of 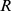. In particular, some global fibre models match or exceed the measured shortening 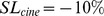. Karadag fibres produce a shortening value smaller than the measured one. The effect of changing form transversely isotropic to orthotropic material law (with the linear sheet model) is shown in Figure 4(c). The orthotropic material law increases the shortening by 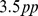 on average. By changing the sheet model to bimodal approximately 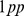 less shortening was obtained.

### Local measures

**Figure 6:**
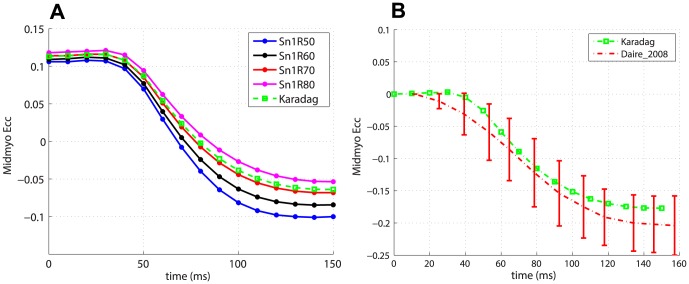
Predicted temporal variation of 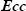. (a) Average midventricular 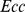 for global fibre models (with 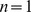) and the Karadag model (b) Comparison of predicted 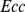 for the simulation with the Karadag fibre model and transverse isotropy and measured 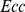 in rat [29].

We considered the circumferential strain 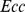, the circumferential-longitudinal shear strain 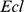 and the circumferential-radial shear strain 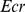, which were uniformly averaged in the basal (upper 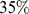 of the left ventricle), midmyocardial (middle 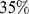 of the left ventricle) and apical sections. We show only the average strain in the midmyocardial region, because it is a good measure of the average behaviour of the left ventricle. In the absence of direct strain data, we compared the predicted traces with experimental results in the literature. Figure 6(a) shows the 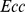 trace for the subset of global fibres models with 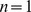 and for the Karadag model in the case of transverse isotropy. The Karadag model has an intermediate behaviour between the fibre models Sn1R70 and Sn1R80, as we have already found for the global measures. Amongst the global fibre models, the magnitude of the strain decreases for increasing values of the angle 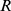. All traces start from a positive value of 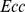 rather than zero, due to the passive expansion performed prior to the electromechanical simulation, which leaves a residual strain component in 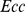. In order to compare predicted 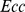 traces with experimental results on 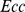 in rat, the study by Daire et al. [29], who employed cine-MRI and Tagged-MRI, was considered. The measurements of 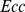 were taken by Daire et al. [29] at fixed percentages of the cardiac cycle, therefore it was necessary to convert this temporal subdivision into a sequence of time points in the range 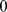
–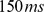 (the duration of our simulations). The data (mean and standard deviation) for the first half of the cardiac cycle, which corresponds approximately to the simulation duration, are shown in red in Figure 6(b). The green line is the 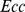 trace for the simulation with the Karadag fibre model and transverse isotropy, after subtraction of the residual strain from the pre-simulation passive expansion. Apart from the inevitable inaccuracy of the conversion from cardiac cycle percentages to the simulation time interval, there is very good agreement between predicted and measured 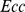.

**Figure 7:**
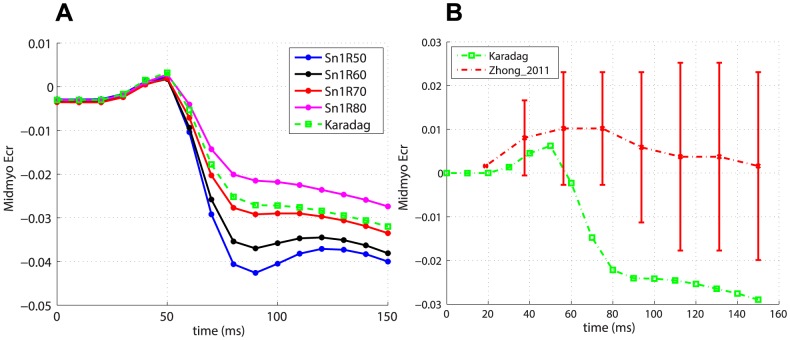
Predicted temporal variation of 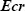. (a) Average 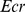 for global fibre models (with 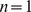) and the Karadag model.(b) Comparison of predicted 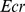 for the simulation with the Karadag fibre model and transverse isotropy and measured 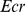 in mouse [31].

**Figure 8:**
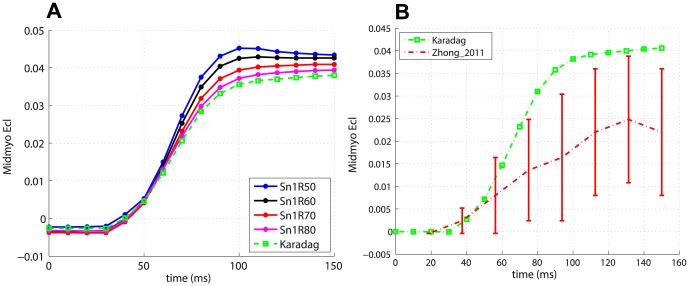
Predicted temporal variation of 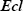. (a) Average midventricular 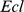 for global fibre models (with 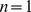) and the Karadag model. (b) Comparison of predicted 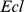 for the simulation with the Karadag fibre model and transverse isotropy and measured 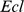 in mouse[31].

Figures 7(a) and 8(a) show the 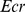 and 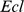 midventricular traces for the subset of global fibres models with 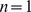 and for the Karadag model in the case of transverse isotropy. With regards to 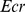, the Karadag model shows an intermediate behaviour with respect to the other fibre models, for which the magnitude of the strain decreases for increasing values of the angle 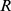. To the best of our knowledge, data on temporal variation of such strains in rat heart was not available in the literature. Instead, we compared predicted shear strains with the measures performed in mouse by Zhong et al. [31]. As with the 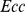, it was necessary to convert from percentages of cardiac cycle to time points in the range 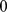
–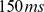. Figures 7(b) and 8(b) show the comparison between data and predicted 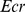 and 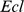, respectively, for the simulation with the Karadag fibre model and transverse isotropy. The agreement for 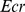 is poorer than for 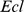. However, in both cases the trends are similar to the experimental ones.

**Figure 9:**
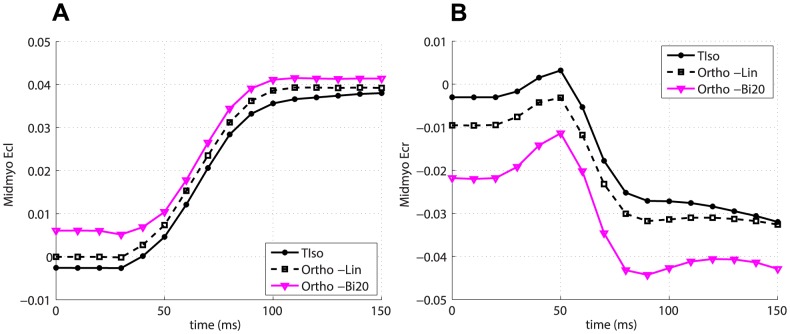
Effect of sheets on Lagrangian strains. Average midmyocardial (a) 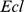 and (b) 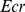 with transverse isotropy, orthotropy with the linear sheet model and orthotropy with bimodal sheet model, in the case of Karadag fibre model.

The effect of sheets was negligible on 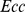, while it was substantial on the shear strains. Changing from transverse isotropy to orthotropy, and within the orthotropic behaviour, from a linear to a bimodal model for the sheets, caused a consistent increase in the magnitude of 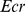 and 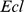, as shown in Figure 9(a) –9(b) for the Karadag fibre model in the midmyocardial region.

## Discussion

In the previous section, results are presented of a systematic computational study into the effect of tissue microstructure on contraction, using a simplified model of rat left ventricle. It consists of a series of electromechanical simulations carried out on a thick-walled truncated ellipsoid, with the aim of quantifying the combined effect of different models for fibre and sheet directions with different tissue passive behaviours during simulated active contraction. Sixteen global fibre models were compared against a subject-specific model. The impact of sheets was also investigated by comparing transversely isotropic and orthotropic material properties as well as two models for the sheet orientation, namely a linear and a bimodal model.

In the development of the model we tried to be as species-specific as possible in all the components, with the exception of the reduced range for the cavity pressure, as described in Methods and further discussed later on. The geometry has dimensions taken from rat cine-MRI scans, the parameters of tissue electrophysiology are based on measurements in rat. Regarding the AP model, we preferred the mouse model of Wang and Sobie [21] over other available rat models for reasons of computational efficiency. Of course, the ventricular activation sequence with simulated apical pacing differs from normal *in vivo* patterns that were present during cine-MRI. However, as we compared pre-activation (diastole) with peak contraction (systole), we believe that this is unlikely to have had a major effect on our findings related to steady-state comparisons. As for the mechanics, there are rat-specific biophysical contraction models. However, we preferred to use the model of Kerckhoffs et al. [24] as it allowed us to adjust its parameters in a controlled manner while retaining a good description of the contractile behaviour of the myocyte. The stiffness parameters of the exponential constitutive law (eqn. (5)) for material orthotropy are taken from a study carried out on canine hearts by Usyk et al. [4]. In their paper Usyk et al. explore the stiffness parameters space in order to study their effect on mechanics, providing a range of variation for each of the parameters. We chose the orthotropic parameter set that showed magnitudes comparable with the values reported for rat in transverse isotropy by Omens et al. [32]. This was necessary because we did not find published orthotropic stiffness parameters values for exponential constitutive laws for rat. We obtained the stiffness parameters for transverse isotropy by averaging the orthotropic ones in the sheet and normal directions. We ensured that the two parameters sets for orthotropy and transverse isotropy had the same magnitudes in order to minimise the effect of parameters magnitude and explore only that of material symmetry.

Various measures were considered, grouped into global and local parameters, in order to study the effect of the fibre model, material law and sheet model on contraction. For the global measures, ejection fraction (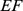), wall thickening (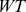) and base-to-apex shortening (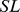) were compared. We chose these measures because they are representative of the main features of cardiac function in the left ventricle. Furthermore, most electromechanical models do not currently predict 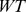 and 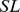 well enough. The global measures are consistently affected by the fibre model and material law, while they show smaller variation when changing the sheet model. The only exception seems to be the downward trend of 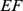 in the case of 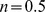 (Figure 3(b)). We believe this is due to an inverse correlation between the magnitude of 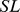 and the rate of increase of 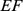. In other words, the more 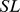 increases in magnitude for a given value of 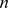 and increasing values of 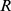, the smaller the increase in 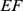. This effect can be seen in Figure 3(b), where the steepness of the 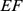 curves decreases consistently going from n = 3 to n = 0.5. This observation suggests that there is a trade-off between shortening and efficiency of the pumping function. Too much shortening is as detrimental to pumping function as elongation. Regarding the fibre models, the subject-specific fibre model does not produce values of the global measures that are notably closer to the measured values than those obtained using the best global fibre models. In particular, among the fibre models, those with low exponent 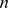 and high torsion angle 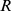 (such as Sn1R70 and Sn1R80) produce values of the global metrics that are comparable or better than those of the subject specific model. These results are encouraging in that they confirm that, in the case of the Streeter-like fibre models (see eqn. (1)), the exponent 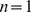 describes fibres in rat left ventricle better than other values, in accordance with previous comparisons of rule-based versus DT-MRI-derived fibres in rat [33]. In general, the predicted values for 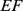 and 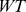, although being at the physiological range ([30],[13]), are lower than the measured ones, while some global fibre models are able to match or exceed the measured value for 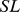. Changing from transversely isotropic to orthotropic material properties (with the linear sheet model) increases the predicted values of the three metrics, but this is not enough to match the measured values for 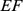 and 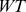. Within the orthotropic setting, no significant effect was seen on 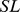, while for 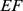 and 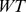 the bimodal model case gave results intermediate between those for transverse isotropy and orthotropy with the linear sheet model.

The Lagrangian strains 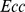, 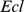 and 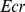 are used as local measures. The Karadag subject-specific model has strain traces close to those of global fibre models with lower exponent 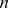 and higher angle 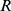. In particular the models Sn1R70 and Sn1R80 are those that in terms of strains give the most similar results to the Karadag model. This confirms our findings in the analysis of the global metrics. Interestingly, the fibre models that produce higher magnitude of strains (that is higher 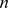 and lower 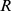) are the same that gave poorest results in terms of the global metrics. Conversely, this means that fibre models such as Karadag, Sn1R70 and Sn1R80 are able to convert smaller strains into more efficient cardiac function. Changing from transversely isotropic to orthotropic material properties has a negligible effect on 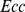, a small effect on 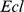, while a more pronounced change was evident in 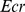. In particular, introducing the bimodal model increases the average magnitude of 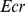 in the midmyocardial region by 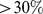 with respect to both transverse isotropy and orthotropy with the linear sheet model. This is as expected since the effect of introducing orthotropy is more likely to have a larger effect on 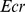 rather than 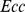. In general, the results show strain traces and magnitudes close to those reported in the literature for rodents [29] [31], especially in the case of 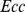, as shown in Figures 6(b), 7(b) and 8(b), for 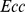, 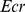 and 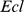 traces, respectively, for the simulation with Karadag fibre model and transverse isotropy.

It is important to discuss the limitations of our model. (a) The use of a simplified geometry: the natural geometrical variability of the left ventricle is not taken into account in this study, including the lack of a neighbouring contracting chamber (right ventricle), or of papillary muscles whose orientation is predominantly axial. Previous studies suggest this might not bring a significant improvement [9], nonetheless, it would be interesting to investigate if the conclusions about the subject-specific vs global fibre models hold also in the case of a subject-specific geometry. (b) The electrical activation pattern: the apical region was chosen for the electrical stimulation in order to mimic the apex-to-base direction of the electrical activation in the left ventricle. This type of activation pattern is consistent, for example, with the type of external stimulation employed in the Langendorff-perfused experiments. However, this is not what happens in the living heart [34]. Evaluating the influence of changes in activation pattern on predicted left ventricular mechanics would be an interesting application of our model. (c) The *ad-hoc* procedure to estimate the pre-P mesh prior to applying the pressure load: this step should be tackled in a more automatic and rigorous approach, by solving an inverse problem. (d) The reduced range of pressures applied in the model: this is probably the most important limitation of the model as pressure is one of the best characterised physiological parameters of the heart. If we compare the results of the preliminary study we carried out using the model without pressure [17] with those presented here, we find similar trends in the global measures for the different fibre models. By introducing pressure in the model, we find that the magnitude of those measures increases significantly on average, meaning that the pressure boundary condition is fundamental for the efficiency of the simulated pumping function, but does not interfere with the effect of the different fibre models on contraction. The use of a reduced pressure range is the result of the best compromise between realistic contraction patterns and physiological pressures in the model. With the current modelling setting, the use of realistic (based on laboratory measurements) material properties in combination with the chosen contraction model forces the pressure to be reduced to avoid non-physiological contraction patterns. The alternative would be to enforce physiological pressures by modifying the material properties (which would no longer be based on experimental measurements) and fully re-parametrizing the contraction model (for which experimental data would not be available). We chose the first approach for several reasons. The first is to reduce the degrees of freedom of the model, setting one parameter (the pressure) rather than a set of mechanical properties. Another is that of consistency with existing literature, which allows us to compare results directly. Still, as mentioned above, this is a major study limitation. As such, it identifies a key direction for further study, reinforcing an important role of quantitative models as a drivers of scientific progress [35], and highlighting that currently available data, implemented mechanisms, and attempted explanations are insufficient to fully reproduce reality. (e) The lack of the transverse angle in the fibre model definitions: this angle regulates the level of crossover of fibres which may be important in a correct prediction of shear strains [16]. (f) Cross-fibre generation of active tension: at the tissue level, the model generates active tension along the fibre direction only. Experimental studies have shown that there is a certain amount of active tension generated in the cross-fibre plane as well [36], and some important electromechanical simulation studies already take this into consideration [4], [16]. In particular, Bovendeerd et al. [16] have shown that including the cross-fibre active tension generation into their model improved the prediction of 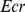. (g) The fibre models considered: the choice of rule-based and subject-specific fibre models analysed was necessarily reduced in order to focus on a specific scientific question. It is important to note that we did not consider asymmetrical fibre angle intervals for the rule-based model, which have been found experimentally [1] and might affect, at least locally, the predicted contraction patterns. However, we expect that their effect would remain within the ranges predicted by the fibre models already examined. Eriksson et al [37], for example, have compared symmetric and asymmetric transmural fibre angles for a linear rule-based fibre model and found generally consistent predictions in the metrics considered. Another important aspect we did not take into account is the variability in subject-specific fibre distribution. Karadag et al [5] report standard deviations of 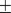 15 degrees for 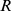 and 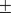 0.25 for 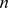. It would be interesting to investigate the inter-subject variability of the Karadag fibre model and its effect on contraction patterns in the future. (h) The sheet orientation models: we used two simplified models of sheet transmural variation, neglecting the great regional variability in terms of sheet patterns that has been found experimentally, and that is thought to give rise to a concertina-like re-arrangement during contraction and relaxation [13]. Overall the impact of sheet inclusion in our study was small, with the exception of changes in 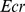. It could be argued that this could be due to the high stiffness in the sheet direction imposed by the constitutive law. However, preliminary tests with different magnitudes of the stiffness parameters did not show such a substantial variation in the global metrics results to make us believe that this problem could be addressed simply by tuning the constitutive law parameters. Furthermore, introducing the more realistic bimodal sheet model did not affect the global metrics in the way we expected. For example, it did not cause a significant increase in the predicted wall-thickening, while it has been shown that this mechanism is directly related to sheets dynamics [14].

To conclude, we developed and tested an electromechanical model and performed a computational study of the impact of tissue microstructure on contraction of the left ventricle in rat. We showed that the model predicts values of global metrics that are within physiological ranges but, except for 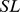, they fall below the values measured on cine-MRI scans. Introducing subject-specific fibre models, as opposed to global fibre models, does not provide a substantial increase in 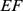 and 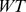 values. The analysis of strains confirmed that the subject-specific fibre model gave predictions similar to linear global fibre models with higher rotation angle. Changing from transverse isotropy to orthotropy in the passive behaviour, therefore introducing the effect of tissue anisotropy due to the sheets, improves the predictions of 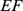 and 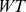, but they still remain lower than the measured values. This means that including the sheet orientation in the model is an important factor, and a more detailed representation of angular re-orientation of sheet material may be required. Therefore, we believe that, within the current modelling framework, the subject-specific approach in fibre definition by itself is not able to compensate for the model limitations that are believed to come from the underestimation of sheet dynamics, and that further investigation of the dynamics of sheet orientation should be carried out and novel approaches developed in order to fully represent its effect on cardiac mechanics.
